# Changes in Galanin Systems in a Rat Model of Post-Traumatic Stress Disorder (PTSD)

**DOI:** 10.1371/journal.pone.0167569

**Published:** 2016-12-01

**Authors:** Karen Barnabas, Lin Zhang, Huiying Wang, Gilbert Kirouac, Maria Vrontakis

**Affiliations:** 1 Faculty of Medicine, University of Manitoba, Winnipeg, Manitoba, Canada; 2 Faculty of Dentistry, University of Manitoba, Winnipeg, Manitoba, Canada; Radboud University Medical Centre, NETHERLANDS

## Abstract

Post-traumatic stress disorder (PTSD) is a chronic syndrome triggered by exposure to trauma and a failure to recover from a normal negative emotional reaction to traumatic stress. The neurobiology of PTSD and the participation of neuropeptides in the neural systems and circuits that control fear and anxiety are not fully understood. The long-term dysregulation of neuropeptide systems contributes to the development of anxiety disorders, including PTSD. The neuropeptide galanin (Gal) and its receptors participate in anxiety-like and depression-related behaviors via the modulation of neuroendocrine and monoaminergic systems. The objective of this research was to investigate how Gal expression changes in the brain of rats 2 weeks after exposure to footshock. Rats exposed to footshocks were subdivided into high responders (HR; immobility>60%) and low responders (LR; immobility<40%) based on immobility elicited by a novel tone one day after exposure. On day 14, rats were anesthetized, and the amygdala, hypothalamus, pituitary and adrenal glands were removed for analysis using real-time polymerase chain reaction (RT-PCR). Gal mRNA levels were increased in the amygdala and hypothalamus of HR compared with the control and LR. In contrast, Gal mRNA levels were decreased in the adrenal and pituitary glands of HR compared with the control and LR. Thus, the differential regulation (dysregulation) of the neuropeptide Gal in these tissues may contribute to anxiety and PTSD development.

## Introduction

The experience of an event that involves intense fear leads to a strong emotional reaction in many individuals. For most individuals, the emotional reaction will dissipate over days or weeks [1–3]; however, it may fail to normalize or may intensify in some individuals, which may lead to the diagnosis of an anxiety disorder referred to as post-traumatic stress disorder (PTSD) [2, 4]. The symptoms of PTSD are believed to reflect stress-induced changes in neurobiological systems and/or an inadequate adaptation of neurobiological systems following exposure to severe stressors [5–7], which underlines the importance of individual differences in the recovery from traumatic fear [8]. Certain neuropeptide systems participate in the behaviors associated with anxiety. It has been shown that long-term dysregulation of these systems contributes to the development of anxiety disorders, including PTSD [9, 10].

There is evidence that Galanin (Gal) and its three general receptor subtypes known as GalR1, GalR2 and Gal R3, play a significant role in anxiety, depression, and stress-like behaviors through modulation of neuroendocrine, serotonergic and noradrenergic systems [10–13]There are also reports suggesting that Gal has antidepressant effects[14] [15]

Gal is a neuropeptide that is widely distributed in the central nervous system of mammals, including humans. This neuropeptide is involved in numerous physiological and behavioral functions, including learning and memory, neuroendocrine control, gastrointestinal motility, feeding and anxiety, through the actions of three receptors (GalR1-3) [16]. In addition, previous genetic studies on humans have indicated that the Gal system is involved in psychiatric disorders, including panic disorders, depression and anxiety [17–19]

The role of Gal in anxiety behaviors depends on the route and site of drug administration and on the intensity of the stress conditions [12]. Intracerebroventricular (icv) Gal administration had anxiolytic-like effects in the rat Vogel conflict test [20], whereas the direct injection of Gal into the amygdala produced the opposite effect in the same task [21]. Consistent with this finding, the intra-amygdala injection of the peptidergic Gal antagonist M40 exerted anxiogenic effects only in animals that were subjected to restraint stress and treated with yohimbine [22]. Using in vivo microdialysis, it was demonstrated that Gal was released in the amygdala by the combination of stress and yohimbine as a result of high noradrenergic activity; however, Gal was not co-released from noradrenergic neurons [23]. The mechanism by which Gal reduces norepinephrine release at locus coeruleus (LC) projections to the amygdala, hypothalamus, and prefrontal cortex may be a direct action of Gal on these brain regions via Gal-synthesizing neurons or the stimulation of Gal receptors in these regions [22, 24]. Furthermore, while various forms of stress (social, exercise, cold, pain, and immobilization) increase prepro-Gal gene expression in the LC, as well as forebrain regions, such as the amygdala and hypothalamus [25–27], other stressors, such as chronic mild stress, have been demonstrated to produce no effect or to decrease Gal mRNA in these same brain regions [28]. This data lead to the conclusion that the effects of Gal on anxiety-like behavior are complex, and whether Gal has anxiolytic or anxiogenic effects appears to depend upon the brain region studied[29]. More recently, it has been demonstrated that Gal is actively involved in the neurobiological response to predator stress and may be associated with recovery processes or resilience to stress exposure [30].

The neurobiological systems that have been implicated in the pathophysiology of PTSD include the hypothalamus-pituitary-adrenal (HPA) axis, as well as various neurotransmitters and neuropeptides involved in a network of brain regions that regulate fear and stress responses, including the amygdala, prefrontal cortex and brainstem nuclei [5].

The aim of this study was to determine whether single foot-shock exposure in rats results in a long-term effect on Gal expression. Stress-associated psychopathologies are often associated with inadequate stress coping and a failure to recover from traumatic life events. Thus, it is important to investigate how stress impacts the long-term expression of Gal and its dysregulation in discrete brain areas involved in fear and anxiety. The correction of abnormal Gal function in fear and anxiety may prove to be a novel target for drug development.

## Methodology

### Animals

Male Sprague-Dawley rats that were 6 weeks of age and weighed 130–160 g (University of Manitoba, Winnipeg, Canada) were paired in cages and maintained in a colony room on a 12-h light-dark cycle (lights on 6 am) under controlled temperature (20–24°C) and humidity (40–70%) conditions for at least 10 days. The rats had free access to food and water and were handled for 5 minutes on alternate days during the adaptation period. The experimental procedures were in compliance with the Canadian Council on Animal Care and were approved by the Research Ethics Review Board of the University of Manitoba.

Foot shock procedure and grouping of animals: The rats were individually transferred to a room that was exclusively dedicated to the delivery of foot shocks. Following a 2-min acclimation period in the foot shock chamber (MED Associates, Vermont, USA), the rats were exposed to foot shocks (5x2 s, 1.5 mA, presented randomly over 3 min with inter-shock periods of 10–50 s) [31, 32]. The rats were maintained in the chamber for an additional 60 s before they were returned to their home cages. The non-shock rats were placed in the chamber for the same amount of time; however, no shock was delivered. The shock chamber was cleaned using 10% alcohol, and the bedding under the grid floor was changed after each rat.

**Fig 1:**
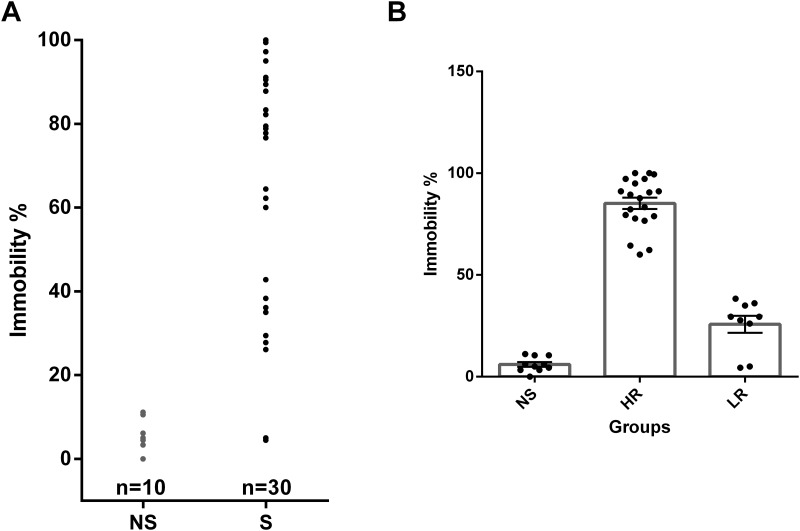
Immobility scores. (A) The individual immobility score for nonshocked (NS) and Shocked (S) rats exposed to a novel tone 24h after the footshock exposure. (B) The mean immobility score for nonshocked (NS) and for shocked rats assigned to low responders (LR) and high responders (HR) groups. The values are expressed as mean ±SEM

On day 2, post-shock fear sensitization was assessed by measuring the amount of freezing expressed in the rats placed in a novel chamber composed of black Plexiglas. The duration of the test was 6 min, which consisted of a 3-min period with background noise and a 3-min period with a novel auditory tone (9 kHz, 75 dB against background noise levels of 45–50 dB). The percentage of freezing, which was defined as a complete lack of body movement with the exception of breathing, was scored for both sessions. The freezing responses to the novel environment were analyzed using one-way analysis of variance (ANOVA) to compare the differences between the non-shock and shock groups (Fig 1). Based on the amount of immobility expressed during the presentation of the novel tone, the shock rats were subdivided into LR (immobility<40%) or HR (immobility>60%) [31, 32]. Humans clearly do not respond homogeneously to potentially traumatic experiences; thus, heterogeneity in animal responses confirms the validity of animal studies. On day 14, the rats were anesthetized with chloral hydrate (600mgéKg, i.p) and perfused with 150ml 0.9%saline. The brain, pituitary and adrenals were quickly removed and put on dry ice for RT-PCR analysis. From the brains amygdala and hypothalamus were dissected on ice using stereomicroscope. Pituitary and adrenals were collected from the same group of animals while we used different group of animals for lateral hypothalamus or amygdala

### Real-time quantitative PCR (qPCR)

To measure the Gal mRNA levels in the dissected tissues, PCR techniques were applied as previously described [33]. Total RNA was extracted from the tissues using the phenol/chloroform extraction protocol provided by Invitrogen (Trizol reagent), purified using an RNeasy^®^ Micro Kit (QIAGEN, Cat # 74004) and stored at -80°C. Total RNA was quantified using a spectrophotometer (Nano-Drop, Cat # ND-1000, Thermo, Fisher Scientific), and 1 mg of total RNA was used to generate single-stranded cDNA. The concentrated total RNA samples were diluted to 0.1 mg/ml at a 10-ml volume. The diluted RNA samples were denatured at 70°C for 10 min and were subsequently added to the reverse transcription (RT) master mix. For RT, the reaction conditions were as follows: 25°C for 10 min, 42°C for 50 min, and 72°C for 15 min with a 0°C hold at the end. The previously generated cDNA templates were diluted with 50 ml RNase-free water. For qPCR, the primer pairs were designed and generated by SABiosciences^™^, QIAGEN; the housekeeping gene GAPDH (glyceraldehyde-3-phosphate dehydrogenase) was used as the internal quantitative control. Each PCR consisted of 12.5 ml RT2 SYBR Green/ROX qPCR MasterMix (Cat # PA-012-24, SABiosciences, QIAGEN), 6.5 ml RNase-free water and 1 ml Primer Mix. PCRs for each gene of interest were run in triplicate using the StepOne^™^ Real-Time PCR System (Applied Biosystems^™^). The PCR cycling program was as follows: 95°C for 10 min and 40 cycles of 95°C for 15 s and 60°C for 1 min. The melt curve program was as follows: 95°C for 15 s, 60°C for 1 min, 65 to 95°C at 2°C/min and 95°C for 12 s. For primer validation, after each PCR cycling program, a default melting program was conducted to ensure that the disassociation curves for each pair of primers contained a single peak, and the agarose gels of the amplified product indicated a single band that corresponded to the predictable amplicon length. To determine the amplification efficiency, a calibration curve was performed prior to the initiation of the experiments with excellent results. Relative quantity (RQ) values were calculated using StepOne^™^ Software v2.1 (Applied Biosystems StepOne^™^). The Gal expression levels in the different tissues were quantitated via comparative reverse transcription real-time PCR. The values of the control glands were set to one.

### Statistics

All data were analyzed using one-way ANOVA followed by Tukey’s multiple comparison test via Graph Pad Prism version 6.0 for Windows. Graph Pad Prism Software (San Diego, California, USA) was used to measure the difference in the Gal levels in the three different PTSD groups: CTL, HR and LR.

## Results

**Fig 2:**
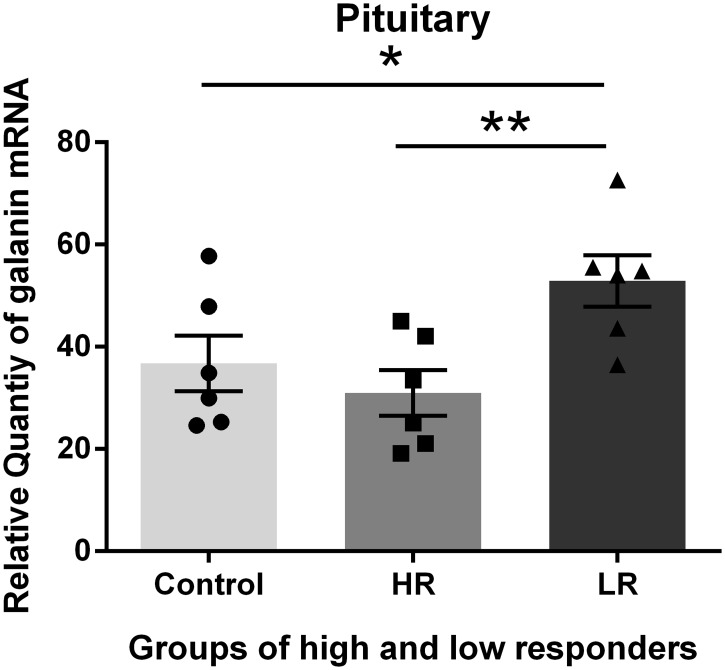
Galanin (Gal) mRNA levels in the Pituitary. Gal mRNA levels in shocked rats subgrouped into low responders (LR) and high responders (HR) at 14 days post-shock. The HR group exhibited a decreased Gal mRNA level compared with the control and LR groups. The difference between the HR and LR was significant. *p <0.05, * *p<0.01. The values are expressed as mean ±SEM

**Fig 3:**
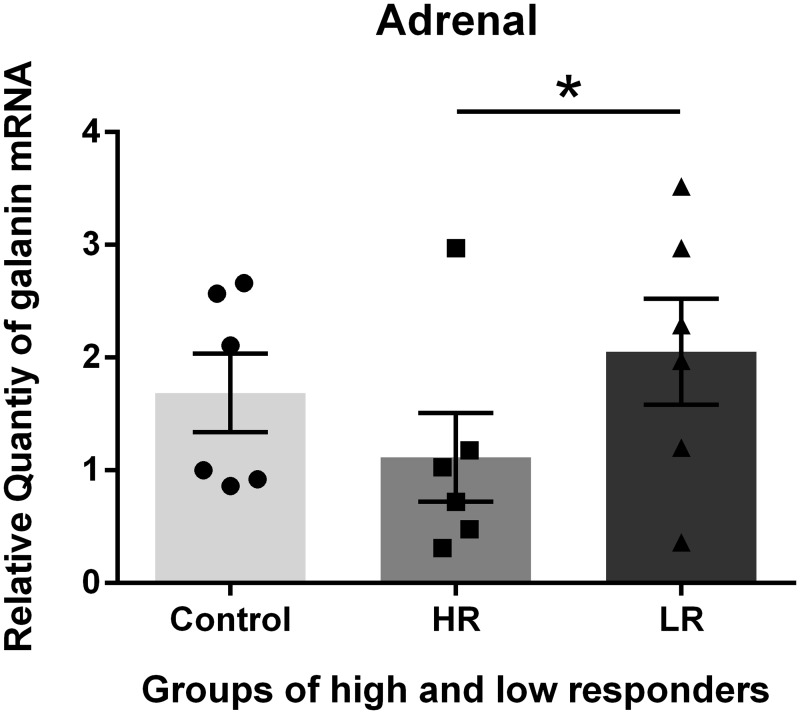
Galanin (Gal) mRNA levels in the Adrenal. Gal mRNA levels in shocked rats subgrouped into low responders (LR) and high responders (HR) at 14 days post-shock. The HR group exhibited decreased Gal mRNA expression compared with the control and LR groups. The difference between the HR and LR was significant. * p<0.05. The values are expressed as mean ±SEM

**Fig 4:**
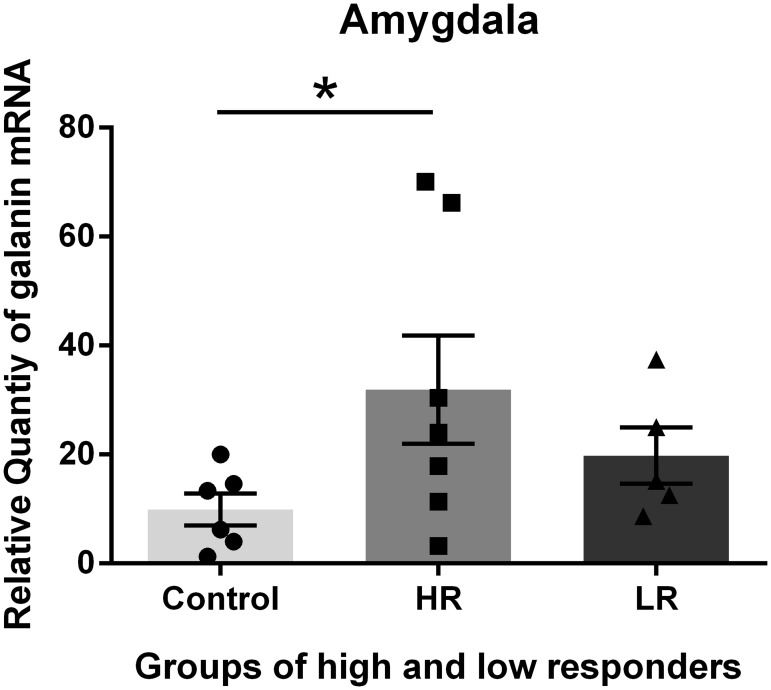
Galanin (Gal) mRNA levels in the Amygdala. Gal mRNA levels in shocked rats subgrouped into low responders (LR) and high responders (HR) at 14 days post-shock. The HR group exhibited an increased Gal mRNA level compared with the control and LR groups. There was no significant difference between the HR and LR. * p<0.05. The values are expressed as mean ±SEM

**Fig 5:**
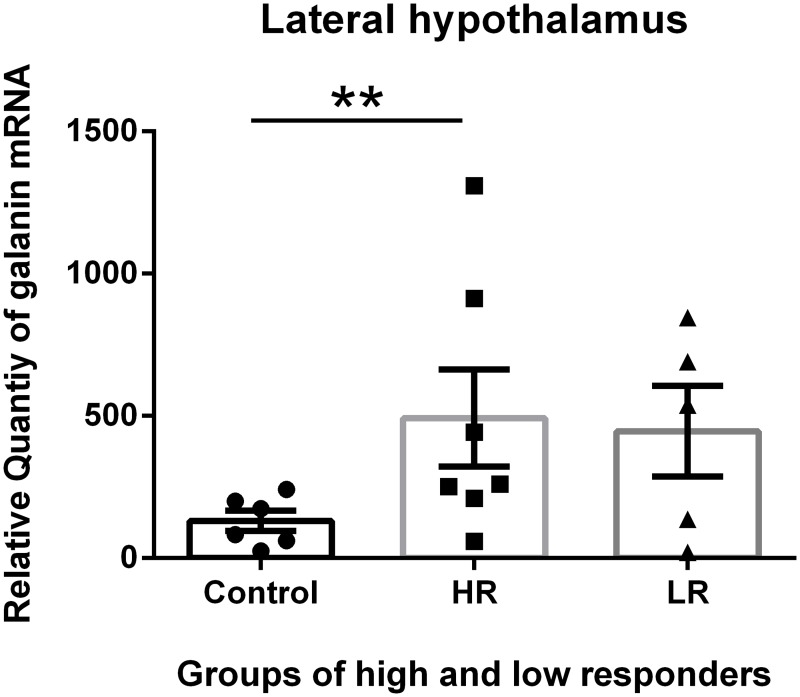
Galanin (Gal) mRNA levels in the Lateral Hypothalamus. Gal mRNA levels in shocked rats subgrouped into low responders (LR) and high responders (HR) at 14 days post-shock. The HR group exhibited an increased Gal mRNA level compared with the controls. There was no significant difference between the HR and LR. * * p<0.01. The values are expressed as mean ±SEM

The exposure of the rats to unpredictable and moderately intense foot shock produced chronic changes in the Gal mRNA levels in the amygdala, hypothalamus, pituitary and adrenal two weeks after the exposure. These changes were different in the 4 tissues examined. The highest abundance of Gal was identified in the hypothalamus, and the lowest abundance was identified in the adrenal glands; thus, the values of Gal expression in the control adrenal glands were set to one for the calculations of the expression levels of Gal in the different tissues via comparative reverse transcription real-time PCR. We divided the shocked rats into HR and LR based on the amount of immobility expressed in response to a novel tone 24 hours after the shock exposure. We subsequently examined the changes in the galaninergic systems in shock-rats 14 days post-shock. HR show more anxiety-related behavioral changes such as decreased exploration in the elevated T-maze at that time (27, 28). The Gal mRNA levels in the pituitary and adrenal glands of the HR were decreased compared with the control and LR. The differences in the Gal mRNA levels between the HR and LR were significant (Figs 2 and 3). In contrast, increased Gal mRNA levels were identified in the amygdala and hypothalamus of the HR compared with the control (non-shocked) and LR. However, the difference in the Gal mRNA levels between the HR and LR was not significant (Figs 4 and 5).

## Discussion

The purpose of the present study was to determine whether the exposure of rats to an acute episode of moderately intense foot shock results in a long-term effect on Gal expression in four brain tissues associated with fear and anxiety.

The present data demonstrate that the exposure of rats to unpredictable and moderately intense foot shocks induced chronic changes in the Gal mRNA levels in the amygdala, hypothalamus, pituitary and adrenal glands two weeks after the exposure. The data also demonstrate that the Gal mRNA levels were different in the HR and LR. It has previously been reported that an early display of pronounced fear following trauma may be associated with long-lasting changes in how the brain processes trauma [31]. Thus, it appears that the alterations in Gal expression (mRNA) in HR and LR following shock occur as molecular adaptations to compensate for stress.

In the experiments presented in this paper, we separated shocked rats into HR and LR based on the amount of immobility expressed in response to a novel tone 24 hours after the shock exposure, and we examined the long-term effect (day14) of the acute episode of foot-shock on Gal expression. It has previously been demonstrated that exposure of rats to moderately intense foot shocks induces long-lasting generalized anxiety in a subset of rats [31, 34–37] and that shock-induced changes in brain peptide activity represent a mechanism for this effect [32]. We identified increased Gal mRNA levels in the amygdala and hypothalamus of the HR compared with the control (non-shocked) and LR. There was no significant difference in the Gal mRNA between the HR and LR.

It has recently been demonstrated in humans that Gal gene expression in the amygdala and hypothalamus is controlled by a region located 45 kb 5’ to the human Gal gene; this pattern is highly conserved among species [38]. This region is not only involved in Gal gene expression; it may also be the only cis-regulatory element required for the tissue-specific expression of the Gal gene in the hypothalamus and amygdala [38]. With respect to depression and anxiety, numerous association studies have linked polymorphisms that surround this Gal locus with mood disorders [17, 39].

In contrast, the Gal mRNA levels in the pituitary and adrenal glands of the HR were decreased compared with the control and LR. There was a significant difference in the Gal mRNA levels between the HR and LR. The cause of the different directions of change in the pituitary and adrenal glands is not clear. Theoretically, reduced Gal expression may contribute to anxiety-like behavior, whereas increased expression may function as a compensatory response to counteract the increased anxiety mediated by other transmitters.

It has been demonstrated that stress increases preprogalanin gene expression in the LC, as well as in forebrain regions, such as the amygdala and hypothalamus [25, 26, 40, 41]. Similarly, although Gal is expressed in the paraventricular nucleus of the hypothalamus with CRF and vasopressin and modulates the responses of the HPA axis to stress, Gal has been demonstrated to increase or decrease the stress-induced activation of the HPA axis depending on the site of administration [42, 43].

Experimental evidence suggests that the release of Gal preferentially occurs under conditions of high norepinephrine activity or stress provocation [44]. It has been demonstrated that the amplification of the noradrenergic response to stress recruits the release of Gal in the amygdala, which subsequently acts to buffer the anxiety-like behavioral response to acute stress [22]. Thus, the net behavioral response to stress is dependent on the overall level of noradrenergic system activation and the resulting balance between NE and Gal neurotransmission. This diverse action of Gal are likely to be the result of the activation of multiple receptors with different signaling pathways [45–47]. GalR1 and GalR2 are expressed throughout the brain [48],while GalR3 is confined to the hypothalamus [49] It has been postulated that the dysregulation of the normal interaction between NE and Gal in the amygdala may contribute to stress-related disorders, such as depression, anxiety and PTSD [22].

In summary, the differential regulation (dysregulation) of Gal expression between HR and LR in the four tissues examined may have contributed to the development of anxiety and PTSD in the HR group. The current findings suggest that the galaninergic system elicits stress modulatory actions, which may serve as a drug target in PTSD. Overall, this study contributes additional evidence that Gal is worthy of further investigation as a novel therapeutic treatment for stress-related disorders.
